# Exploring the Impact of Cognitive Dysfunction During Recurrent Depression in a Sample of Mid‐to‐Older Age British South Asians: A Qualitative Study

**DOI:** 10.1111/jpm.13113

**Published:** 2024-09-24

**Authors:** Amirah Akhtar, Shabana Shafiq, Sahdia Parveen, Emmanuel Nwofe, Karen Windle

**Affiliations:** ^1^ Centre for Applied Dementia Studies University of Bradford Bradford UK

**Keywords:** cognitive functioning, depression, functional impairment, lived experience, qualitative, recovery

## Abstract

**Introduction:**

Depression is a major public health issue, increasing the risk of comorbidities. Some people with depression experience cognitive dysfunction, which can persist even after symptomatic recovery. British South Asians are at greater risk of developing depression and are less likely to seek treatment. It is important to understand their experience of subjective cognitive dysfunction in depression and how best to support them.

**Aims:**

This study explored subjective experience of cognitive dysfunction during recurrent depression, in a sample of 12 British South Asians aged between 45 and 60 years.

**Methods:**

We conducted semi‐structured interviews to explore cognitive dysfunction during recurrent depression. We analysed the data using thematic analysis.

**Results:**

Difficulties in attention and concentration resulted in lower quality of social relationships, including not feeling present and social isolation. Learning new information was difficult, thus impacting productivity. Participants found it difficult to engage in enjoyable activities that promoted brain health. The emotional, physical and spiritual impact negatively impacted on quality of life.

**Discussion:**

Cognitive strategies used in therapies could improve brain health and functional recovery in people living with depression.

**Implications:**

Mental health nurses play a pivotal role in providing culturally appropriate information and strategies for managing cognitive dysfunction in recurrent depression.

**Summary**

- What Is Known on the Subject?
  ○ Depression is a public health issue, increasing the risk of comorbidity. When depression recovery is not achieved or maintained, there is a greater risk of recurrent episodes of depression. This can lead to suicide risk, recurrent hospitalisations and greater morbidity.
  ○ Even when mood symptoms resolve, functional impairments may persist.
- What the Paper Adds to Existing Knowledge?
  ○ This paper provides an in‐depth qualitative understanding of the subjective experience of cognitive dysfunction during episodes of recurrent depression, in the British South Asian population.
  ○ It identifies the impact of cognitive dysfunction on psychosocial functioning and the difficulties in taking part in enjoyable, brain health‐promoting activities.
  ○ The impact on quality of life and the emotional, physical and spiritual burden associated with cognitive dysfunction is reported.
- How Could the Findings Be Used to Influence Practice, Education, Research, and Policy?
  ○ Mental health nurses need the correct knowledge and training to support people of South Asian background, in providing culturally appropriate support to manage cognitive difficulties during depressive episodes.
  ○ Mental health nurses, wider multidisciplinary team and people with lived experience work together to codesign interventions that promote not only symptomatic but also functional recovery.

## 1 Introduction

Depression is the third leading cause of disability worldwide (World Health Organisation 2017), impacting quality of life of the person living with depression and their families, as well as posing a significant burden on health care systems (James et al. 2018; Kyu et al. 2018). In the United Kingdom, the prevalence of depression has been shown to range from 11.3% for mild depression and 3.3% for severe cases (De la Torre et al. 2021). British South Asians experience depression at a higher rate compared to their White counterparts. Williams et al. (2015) reported a prevalence rate of 15.5% for people of South Asian background compared to 9.7% for individuals of White background. This was attributed to socio‐economic disadvantage and increased risk of chronic, comorbid conditions such as diabetes and cardiovascular disease in British South Asian communities.

Depression is linked with increased risk of morbidity and is reported as 1 of the 12 modifiable risk factors for dementia by the Lancet Commission (Livingston et al. 2020). The nature of depression means that people often experience loss of interest in enjoyable activities, sleep and appetite disturbances. These symptoms can lead to a decrease in self‐care behaviours, impacting a person's health. Studies have shown depression to increase the risk of conditions such as diabetes (Yu et al. 2015) and cardiovascular disease (Li et al. 2022).

The relationship between depression and dementia is complex and the evidence remains mixed. Some studies suggest early‐life depression as a risk factor for dementia and late‐life depression as a prodrome of dementia (Byers and Yaffe 2011; Kuring, Mathias, and Ward 2020; Xu et al. 2023). Several longitudinal studies have found a link between depression, declining cognition and increased risk of dementia (Gatchel et al. 2019; Almeida et al. 2017). A significant number of people living with depression also experience cognitive dysfunction, impacting their memory, attention and concentration. This can impact occupational and psychosocial functioning (Fehnel et al. 2016; Gonda et al. 2015).

In some cases, cognitive dysfunction persists even after symptomatic recovery from depression. In a prospective cohort study, Conradi, Ormel, and De Jonge (2011) found cognitive symptoms to be prevalent at around 85%–94% across the course of depression and between 39% and 44% during remission. A meta‐analysis by Semkovska et al. (2019) suggested cognitive dysfunction in areas of selective attention, working memory and long‐term memory persisted after remission of depression. The presence of cognitive dysfunction after symptomatic recovery from depression can increase the risk of relapse. Recurrent depression across the life course has been associated with increased risk of suicide, hospitalisation and the development of long‐term conditions such as dementia (Lee et al. 2021; Dotson, Beydoun, and Zonderman 2010; Kessing and Andersen 2004).

Airaksinen et al. (2004) examined cognitive functioning in population‐based samples to determine whether cognitive function varies as a function of depression subgroup, in patients aged 20–64 years. Four groups were compared including major depressive disorder, dysthymia, mixed anxiety‐depression and minor depression with non‐depressed controls. Participants across the depressed groups exhibited impairments in episodic memory and mental flexibility. The major depression and mixed anxiety‐depressive disorder groups exhibited significant memory dysfunction. Participants with dysthymia showed pronounced difficulties in mental flexibility. Minor depression did not affect cognitive performance. Singh‐Manoux et al. (2010) examined the association between depressive symptoms and cognitive dysfunction to better understand dementia risk, using data from the Whitehall II cohort, a longitudinal study originally designed to examine health inequalities among British civil servants from 1985 until 2004 (Marmot et al. 1991). Data from the General Health Questionnaire, a battery of cognitive tests and the Mini Mental State Examination (MMSE) suggested that individuals experiencing persistent depressive symptoms were at increased risk of developing cognitive dysfunction in late midlife, thus increasing the risk of developing dementia.

It is also important to understand what impact cognitive dysfunction in depression has on people's daily lives to develop more targeted interventions that promote functional recovery, in addition to symptomatic recovery. Qualitative studies focusing on the lived experience of depression and subjective cognitive dysfunction are currently lacking. Crowe et al. (2020) conducted a qualitative study in New Zealand, in which depressed participants recalled their subjective cognitive dysfunction. Key experiences included feeling stuck physically and mentally, unable to get up and do simple tasks. The participants also reported being pre‐occupied with their own thoughts, including rumination and zoning out during conversations. Fehnel et al. (2016) found that cognitive symptoms of depression affected productivity at work. Cognitive symptoms also had a social impact as participants struggled to make or remember plans.

The study by Crowe et al. (2020) and Fehnel et al. (2016) emphasised the importance of recognising cognitive dysfunction in depression to promote functional recovery. However, links to brain health or dementia prevention were not made. In the United Kingdom, approximately 850,000 people are diagnosed with dementia, of which 25,000 are from minority ethnic backgrounds (Alzheimer's Society 2024). This number is predicted to rise to 50,000 by 2026 and 172,000 by 2051. This indicates a sevenfold increase for minority ethnic populations, compared to a twofold increase in White British populations (Wohland et al. 2010). Thus, minority ethnic populations such as those of British South Asian heritage are more likely to be affected by both depression and dementia.

### 1.1 Aim

Whilst symptomatic recovery is important in depression, functional recovery is sometimes overlooked (Crowe 2017). It is imperative, therefore, to study the impact of cognitive dysfunction from the perspectives of people living with recurrent depression, to understand how it impacts their daily life.

The negative impact of cognitive dysfunction during and sometimes postdepression makes it an important outcome to monitor. Availability of this data could play a role in dementia prevention and the promotion of brain health. The British South Asian population face multiple barriers in accessing not only mental health support but also receiving a diagnosis of dementia (Harwood et al. 2023; Blakemore et al. 2018). A subjective understanding of patients' experience of depression and cognitive symptoms can illuminate difficulties in social and occupational functioning, compared to objective measures (Ott et al. 2016). A qualitative approach allows for an in‐depth understanding of experiences. The aim of this study was to, therefore, explore subjective experiences of cognitive dysfunction during recurrent depression, in a sample of British South Asians aged between 45 and 60 years.

## 2 Materials and Methods

This qualitative, descriptive study utilised semi‐structured interviews as its methodology, to explore experiences of cognitive dysfunction during depression. Ethics approval was granted by the Chair of the Humanities, Social and Health Sciences Research Ethics Panel at the University of Bradford, reference number E1150.

### 2.1 Participant Recruitment

Participants were recruited via purposive sampling. Inclusion criteria included being of British South Asian background, aged between 45 and 60 years, with experience of recurrent depression and subjective cognitive dysfunction. Exclusion criteria included having a diagnosis of mild cognitive impairment or dementia. Studies have demonstrated low uptake of NHS Talking Therapies among ethnic minority groups, due to inequalities in the referral and assessment process (Harwood et al. 2023). Finding potential participants via the NHS thus may have risked lower levels of recruitment. The first author utilised their existing links with community mental health organisations, where it was evident that many service users attended, to maximise recruitment. The study was also advertised on social media. Interested participants contacted the researchers and were sent a participant information sheet. A total of 12 participants took part in this study, see Table 1 for participant characteristics. Informed consent was obtained from all participants prior to the interviews being conducted for their data to be used in research. Participants also completed a demographics form.

**TABLE 1:** Participant characteristics.

Participant no	Gender	Age	Ethnicity	Education level	Occupation	Recurrent depression (years)	Treatment	Language of interview
1	Male	50	Pakistani	Post graduate diploma	Project manager	5	Non‐pharmacological	English
2	Female	48	Indian	BA (Hons)	Project manager	2	Non‐pharmacological	English
3	Male	60	Pakistani	No formal qualifications	Unemployed	25	Pharmacological and non‐pharmacological	Pothwari
4	Female	56	Pakistani	GCSE	Unemployed	5	Pharmacological and non‐pharmacological	Punjabi
5	Female	58	Pakistani	GCSE	Housewife	17	Pharmacological and non‐pharmacological	English
6	Female	60	Pakistani	No formal qualifications	Housewife	6	Pharmacological and non‐pharmacological	Pothwari
7	Female	53	Pakistani	GCSE	Daytime and Activities lead (Community centre)	3	Pharmacological and non‐pharmacological	English
8	Female	46	Indian	NVQ	Unemployed	31	Pharmacological and non‐pharmacological	English
9	Male	48	Pakistani	No formal qualifications	Unemployed	20	Pharmacological and non‐pharmacological	English
10	Female	49	Pakistani	MA	Unemployed	22	Pharmacological and non‐pharmacological	English
11	Male	58	Pakistani	GCSE equivalent	Unemployed	6	Pharmacological	Punjabi
12	Female	60	Pakistani	GCSE equivalent	Housewife	31	Pharmacological and non‐pharmacological	Punjabi

### 2.2 Data Collection

Interviews followed a topic guide and questions were developed based on existing literature as well as being based on discussions with the wider research team. All interviews were conducted by the first author who is bilingual, of South Asian background and Muslim. Seven interviews were conducted in English, two interviews were conducted in the Pothwari dialect and three interviews were conducted in Punjabi. For participants who did not speak English, the consent form and information sheet were verbally translated. Two interviews were conducted via Microsoft Teams, three in participant homes and seven over the phone. All interviews were audio‐recorded and transcribed verbatim. All of the English interviews were transcribed via an external transcription company. Two Punjabi interviews were translated into English via an external transcription company. The remaining three non‐English interviews were transcribed and translated by a bilingual researcher (SS). Transcripts were checked for accuracy and anonymised by the first author before analysis. The interviews varied from 30 to 60 min in length. All participants were debriefed at the end of the interview and received a £20 shopping voucher as a thank‐you for their time. Data collection continued until theoretical saturation, whereby no further new concepts were discussed by participants (Braun and Clarke 2021). Questions explored participants' experience of depression and of subjective cognitive dysfunction, probing for any specific challenges around memory, attention, concentration and how this impacted social and occupational functioning.

### 2.3 Data Analysis

Data were analysed using thematic analysis (Braun and Clarke 2021). Transcripts were uploaded into Nvivo 12 Pro software for analysis. A deductive analytical approach was taken with the development of a preliminary coding framework using concepts from the interview questions. This included codes related to depression, subjective cognitive dysfunction and any impact on occupational and social functioning. Two researchers independently and systematically coded two of the transcripts and discussed the codes and themes. There was an element of inductive coding if participants mentioned something relevant not captured by the preexisting framework. One researcher continued coding the data using the revised framework. Codes were then organised into themes reflecting the research questions.

### 2.4 Patient and Public Involvement

Organisations providing support for people living with depression were involved in the development of this study, this included providing suggestions of what to include in the topic guide.

## 3 Results

Two master themes were developed: the first theme captured the impact of cognitive dysfunction on psychosocial functioning and brain health. The second master theme captures the impact of cognitive dysfunction on quality of life.

### 3.1 Master Theme: Psychosocial Functioning and Brain Health

This theme captures how challenges with cognitive dysfunction during recurrent depression impacted people's social relationships and prevented them from taking part in brain health activities.

#### 3.1.1 Feeling Misunderstood

Difficulties in maintaining concentration and attention during social interactions were found to affect personal relationships. Participants were left feeling alone, isolated and misunderstood. In the quote below, the micro dialogue emphasises that experiences of depression were labelled as not valid by friends, in comparison to other struggles or illnesses people may face. The impact of depression is highlighted as something which takes control over your life, as you no longer have the motivation nor desire to take part in enjoyable activities.

I lost all my friends. I've got no friends anymore because they don't understand me. They just look at me and they say ‘oh, but people have got other problems out there. People are worse’. And I'm not even saying that. Of course, people have got worse things out there. But when depression takes over your life, you don't enjoy anything. (Participant 8)

The intensity of feeling misunderstood is reported, resulting in feelings of isolation, loneliness and a desire to no longer be in the present reality because of depression and cognitive dysfunction.

Yes, and I have a feeling, like, the whole world, everyone's against me. I have a feeling like that. I'm alone in this world. I feel like running away from this world. (Participant 9)

#### 3.1.2 Not Feeling Present

Participants reported not feeling present during conversations due to their inability to concentrate and meaningfully engage, leading to poorer quality of interactions. This caused the person living with depression to feel negatively and self‐conscious regarding how they are being perceived by others, impacting their social relationships.

If I respond with non‐committal noises like ‘uh’ or ‘yeah,’ people accuse me of not listening. I often have to apologize and admit I missed what was said. I reflect on it and feel disheartened, worrying that people might think I'm ignoring them when they come to speak with me. (Participant 12)

I find myself just don't know quite… it's not even quite daydreaming but just kind of zoning out. Yeah, I just find myself zoning out but to be honest. (Participant 10)

#### 3.1.3 Reduced Participation in Brain Health Activities

The inability to concentrate and lack of motivation during depressive episodes led participants to no longer take part in enjoyable activities. Participation in activities which promote cognitive reserve and brain health such as reading and engaging in meaningful social interactions was reduced.

Before I used to read the newspaper, Urdu newspaper, I used to enjoy reading that. But now it's been a few years, I can't even concentrate or focus on reading the newspaper. I used to watch the tv before and now I can't concentrate on the TV either. So, I've stopped doing a lot of these things. (Participant 3)

I've lost interest in engaging in activities or even communicating with others or even talk to somebody over phone. My preference is to wander outside aimlessly. I struggle with using my phone, frequently forgetting why I picked it up in the first place. All these things happen to me. (Participant 11)

#### 3.1.4 Occupational Functioning

Only 3 out of 12 participants in this study were in paid employment, with three being housewives and six being unemployed. Reasons for unemployment included living with depression and other long‐term health conditions such as chronic fatigue, stroke, obsessive compulsive disorder and fibromyalgia. Participants in paid employment reported how cognitive dysfunction during depression made it difficult for them to learn new skills at work. This was attributed to difficulties around attention and concentration. Attempts were made at managing this by using strategies such as note‐taking to re‐enforce new information.

I will forget things more regularly. I've been in this job for the last three months and I can struggle to concentrate sometimes and learning new things. Other than actually going and training and learning and focusing on the learning or listening to the learning I become distracted. I can become disengaged, or if it's like watching a learning video, I'd rather put it off. (Participant 1)

If I'm going through anxiety or depression or a bad, bad day, or bad night, then it's very difficult to concentrate, yes. But there's a pattern. Do you know, when I'm working, maybe because I've been working at this place for so long, it's in my mind fitted. I can do all right here, because I've got so much practice. But when something new comes up, it takes time. (Participant 7)

In terms of the zoning out in the meeting, you manage that by, you know, making notes to yourself. And when you're in a clearer mindset, then you go back to it. (Participant 2)

### 3.2 Master Theme: Impact of Subjective Cognitive Dysfunction on Quality of Life

This theme captures how cognitive dysfunction impacted on quality of life, particularly in these three areas of wellbeing: emotional, spiritual and physical.

#### 3.2.1 Emotional Impact

Participants reported forgetfulness, from forgetting appointments resulting in further stress and potentially dealing with the consequences of being struck off the patient register. Dealing with the impact of forgetfulness took considerable energy from participants, leading to negative self‐representations.

I often forget appointments, why I've entered a room, such as going to the kitchen for water, or I might suddenly become irritable. I feel anger within myself. (Participant 12)

Furthermore, participants highlighted just how much cognitive challenges impact quality of life, as day‐to‐day life is consumed by anxiety, anger and no enjoyment in life.

I get sick of life, what else can you feel? You become frustrated with yourself and the things you can no longer do. I get angry, I feel anxious, just get sick of living. (Participant 3)

The consequences of forgetting to do tasks in professional settings are demonstrated and a vicious cycle is described whereby the participants feel that they are always behind schedule. This results in the inability to relax as sleep is affected, and a reported increase in depression symptoms. In this case, symptomatic and functional recovery is likely to be comprised as a result of the cognitive challenges experienced.

With my forgetfulness, or I forgot to do something, and I feel pushed back, then a huge amount of depression comes in me, where I feel like I've not done this in time, and now I won't be able to finish my work off in time, you know? It just… Even though weekends I don't sleep in. (Participant 7)

#### 3.2.2 Impact on Spirituality/Religion

Most participants who reported cognitive dysfunction as impacting their ability to perform prayers were of Muslim background. This included difficulty in the five daily prayers, which requires the individual to memorise verses and to perform the prayer in a particular order and sequence. The quotes below suggest the importance of praying properly, if in doubt participants repeated prayers until satisfied, although this led to extra energy being expended resulting in exhaustion, both physical and mental.

Sometimes, I get up to pray. However, after performing my obligatory prayers, I find myself uncertain if I've completed them, leading me to repeat the prayers multiple times. (Participant 11)

My concern about incorrectly performing prayers, especially missing a rakat, leads me to repeat them. This issue, while common, can be particularly severe for those of us dealing with depression. It is indeed exhausting. Nevertheless, fulfilling the obligation of prayer is crucial, so I adapt by sitting if standing becomes too difficult. (Participant 12)

Despite great attempts at memorisation, the participant was unable to memorise, resulting in feelings of guilt, shame and embarrassment. Identifying as Muslim, the participant in extract 9 indicates a sense of responsibility to perform funeral prayers for family members, being unable to do so was emotionally distressing.

I struggle with this and even for example, a major concern for me is at the age of 50, I still don't know the funeral prayer, the Janaza prayer, and I couldn't remember that for my daughter. I couldn't remember it for my father, and that is a that gives me a sense of shame and embarrassment that no matter how much I've tried, I've learned about three quarters. (Participant 1)

#### 3.2.3 Physical Impact

Participants reported how their depression and cognitive dysfunction led to feelings of tasks taking longer to complete, resulting in greater energy expenditure. The stress related to this reportedly led to somatic symptoms such as the experience of headaches, increased blood pressure and bodily aches and pains, as demonstrated in the quotes below.

It takes a lot of energy, yes. Then it's like I feel so bad. I feel like my shoulders are hurting. I get headaches, real headaches. I start having… I think my blood pressure goes high, and my eyes start burning. I just feel tired and yawning. It's frustrating when that happens. (Participant 7)

Realizing I've forgotten an appointment brings on a lot of stress. This stress then leads to migraines. I experience sharp pains at the back of my head right above the neck due to the tension caused by constantly forgetting things. It starts slowly and consequently becomes a stress. So, it happens overthinking and most often I forget and experience lot of tension. (Participant 11)

## 4 Discussion

All participants in this study reported subjective cognitive dysfunction during episodes of recurrent depression. This included experiencing challenges around memory, attention and concentration, which made day‐to‐day functioning a struggle, impacting overall quality of life. The inability to maintain attention and concentration during social interactions negatively impacted social relationships. Participants reported struggles around feeling present during interactions and how they were perceived as being rude or uninterested. Due to this reason, they were more likely to socially isolate themselves from others, which is another risk factor for dementia. This finding is in line with previous research which found persistent cognitive difficulties to be associated with psychosocial impairment, poorer quality of life and increased risk of relapse or reaching recovery (Matcham et al. 2023; Atique‐Ur‐Rehman and Neill 2019; Baune and Renger 2014; Ebert et al. 2017; Martinez‐Aran et al. 2009).

One of the symptoms of depression is a lack of motivation to partake in enjoyable activities, such as those that promote cognitive reserve (e.g., reading). This is perhaps beyond just a mood feature of depression and may also be exacerbated by subjective experiences of cognitive dysfunction. For example, issues with attention and concentration may make reading and comprehending texts difficult. The experience of recurrent depression and lack of participation in brain training activities may lead to poorer brain health overall. Currently, there is no strong evidence base for brain training and dementia risk reduction. Mowszowski, Batchelor, and Naismith (2010) conducted a review which evaluated cognitive training as a preventative tool in healthy older adults, at‐risk individuals and in the clinical population. Cognitive training was found to improve cognition in healthy older adults and those with mild cognitive impairment. However, the evidence for cognitive training in relation to Alzheimers disease was mixed. One of the main issues for this included diverse methodologies with varying sample sizes and a lack of data on functional outcomes. A systematic review by Gates et al. (2011) found cognitive exercises to produce moderate to large beneficial effects on memory‐related outcomes for adults at risk of dementia. However, one of the main gaps in the evidence base is the low number of high‐quality randomised controlled trials.

The Alzheimer's Society (2024) (a dementia charity in the United Kingdom) recommends focusing on the 12 modifiable risk factors to reduce your risk of developing dementia. This includes having a well‐balanced healthy diet, managing health conditions such as diabetes, being physically active, socially active and taking care of your mental health. However, the present study found that for people living with depression and subjective cognitive dysfunction, participating in brain health‐promoting activities can be difficult. Cognitive dysfunction was reported to impact overall quality of life. Three key areas were identified, which were the emotional, spiritual and physical impact of cognitive dysfunction in recurrent depression. Emotionally, participants felt frustrated and angry as they struggled to navigate through daily life, from completing household chores to making and managing appointments. The majority of participants in this study were of Muslim background and reported struggles during prayer times, such as remembering the sequence of prayer. This led to not only the emotional burden of mental exhaustion, frustration and religious/spiritual guilt but also a physical impact, as continuously praying or doing ablution led to joint pains. In the present study, 50% of the sample were unemployed due to depression and various other health conditions. The small number of individuals who were employed encountered challenges in their working lives due to subjective cognitive dysfunction in depression.

Without appropriate support which considers both symptomatic and functional recovery, people may be prevented from entering or remaining within the workforce. A recent report by the Mental Health Foundation and London School of Economics (McDaid and Park 2022) reported mental health problems cost the UK economy £118 billion, of which 72% is related to loss of productivity. These statistics indicate an economic need for effective preventive measures.

### 4.1 Implications for Practice

Currently, there is a lack of recognition from clinicians of the importance of focusing on cognitive symptoms in the treatment of depression (McAllister‐Williams et al. 2017). Mental health nurses play an important role in supporting people living with recurrent depression to manage cognitive dysfunction and to promote functional recovery. The findings of this study can support mental health nurses in offering culturally appropriate support and advice for the South Asian population. This includes raising awareness of cognitive dysfunction in depression and how this is a functional symptom, as opposed to a negative personal attribute particularly as participants in this study felt negatively about themselves when they struggled with their religious prayers.

The small but growing field of cognitive remediation (CR) therapy for depression has shown some promising results. CR aims to improve concentration, memory, planning and thinking skills through talking and activities. It is often considered under the same umbrella as ‘brain training’ which can include computerised or application‐based games, designed to keep the mind active, sometimes promoted as protection from cognitive decline. Nguyen, Murphy, and Andrews (2022) conducted a meta‐analysis of 43 studies to evaluate the efficacy of commercial brain training programmes in health older adults and older adults with mild cognitive impairment. The review found insufficient empirical evidence to support the idea that commercial braining training programmes can improve cognition or everyday functioning. In contrast, CR is considered a holistic form of therapy which includes a therapist supporting the individual to help mitigate the impact of cognitive challenges. This is achieved through metacognition, and developing awareness and approaches to manage cognitive challenges in daily life (Wykes, Bowie, and Cella 2024). A systematic review and meta‐analysis of eight studies by Therond et al. (2021) explored the efficacy of cognitive remediation on global cognition and six cognitive domains. Cognitive remediation was found to improve global cognition, verbal memory, attention/processing speed, working memory and executive functioning. Culturally adapting cognitive remediation therapy could be beneficial to the South Asian population, who in this study reported challenges within the above‐mentioned cognitive domains. Targeting these cognitive domains could be a focus for mental health nursing in promoting functioning recovery in recurrent depression, reducing the risk of relapse.

Whilst the focus of this paper has been on addressing depression early in life as a preventative measure to negate memory issues/dementia, it is important to also consider the bi‐directional relationship between depression and dementia. Anti‐depressant use in people living with depression and dementia has shown to not always be effective and is not recommended as the first line of treatment (Costello, Roiser, and Howard 2023). A person‐centred approach should be taken to assess whether it is appropriate for the person living with dementia to receive psychological therapies. The Alzheimer's Society (2023) recommends ways in which to support people living with depression and dementia. This includes similar advice given for dementia prevention, such as keeping socially active, physically active, taking part in enjoyable activities and having a healthy‐balanced diet. Of course, these are more challenging for a person with depression and dementia.

It is important for mental health nurses to feel supported in working with this population, through training and resources to help implement such advice. Cooper et al. (2024) conducted a cluster‐randomised single‐blind trial of a dementia training and support intervention for UK homecare agencies, known as NIDUS‐professional. This was a manualised intervention, to reduce home care worker strain and improve quality of life of clients, the results indicated NIDUS‐professional to be potentially feasible and acceptable and received well by homecare workers (Kelleher et al. 2024). One of the manualised sessions included supporting people to stay active and involved in meaningful activities. Whilst a full‐scale trial is needed, interventions like NIDUS‐professional could also be beneficial in supporting mental health nurses in managing depression in dementia.

### 4.2 Limitations

A limitation of this study is that the sample lacked diversity in terms of ethnic and religious background. The majority of participants in this study were of Pakistani and Muslim background. Thus, the experiences outlined may not be representative of all British South Asians. Whilst participants expressed how subjective cognitive dysfunction impacted their day‐to‐day life, participants did not directly link their experiences with brain health. This might be due to lack of awareness of depression as a risk factor for dementia and overall brain health. Further research is required to address this gap.

## 5 Conclusion

Currently, cognitive dysfunction is not assessed or monitored during the treatment of depression. Whilst symptoms of cognitive dysfunction may not always be directly reported by service users during their referral for psychological therapies, it no doubt has a significant impact on their psycho‐social and occupational functioning as the findings of this study demonstrate. One of the elements which make depression a complex risk factor across conditions is its bi‐directional relationship and causality. By managing symptomatic as well as functional recovery via improved cognition, it is possible to improve overall quality of life and promote brain health across the life course. The implementation of cognitive strategies in therapies may support people living with depression and improve symptomatic and functional recovery. Measuring cognition during the treatment of depression may contribute towards our understanding of depression as a risk factor for brain health and long‐term conditions such as dementia. The availability of this data may allow us to understand whether depressed individuals with cognitive dysfunction earlier in life are at greater risk of developing memory issues later in life.

## 6 Relevance Statement

Currently, the focus on treatment for depression is on symptomatic recovery, whilst cognitive dysfunction and functional recovery is not a priority. Mental health nurses particularly in the community, can play a role in providing culturally appropriate support for people of South Asian background living with recurrent depression and cognitive dysfunction.

## Ethics Statement

All participants provided informed consent, and ethical approval was provided by Chair of the Humanities, Social and Health Sciences Research Ethics Panel at the University of Bradford, reference number E1150.

## Conflicts of Interest

The authors declare no conflicts of interest.

## Data Availability

The data that support the findings of this study are available from the corresponding author upon reasonable request.
